# Extraction and Evaluation of Bioactive Compounds from Date (*Phoenix dactylifera*) Seed Using Supercritical and Subcritical CO_2_ Techniques

**DOI:** 10.3390/foods11121806

**Published:** 2022-06-19

**Authors:** Kashif Ghafoor, Md. Zaidul Islam Sarker, Fahad Y. Al-Juhaimi, Elfadil E. Babiker, Mohammed S. Alkaltham, Abdullah K. Almubarak

**Affiliations:** 1 Department of Food Science and Nutrition, College of Food and Agricultural Sciences, King Saud University, Riyadh 11451, Saudi Arabia; faljuhaimi@ksu.edu.sa (F.Y.A.-J.); ebabiker.c@ksu.edu.sa (E.E.B.); malkaltham@ksu.edu.sa (M.S.A.); aalmubarak252@gmail.com (A.K.A.); 2 Food Science Program, Cooperative Research, Extension and Education Services, Northern Marianas College, Saipan, MP 96950, USA; mdzaidul.sarker@marianas.edu

**Keywords:** *Phoenix dactylifera*, date fruit seed, supercritical fluid, subcritical CO_2_, phenolics, carotenoids, anthocyanins, flavonoids, antioxidant properties, green extraction methods

## Abstract

Date (*Phoenix dactylifera*) seed is a potential source of natural antioxidants, and the use of innovative green and low temperature antioxidant recovery techniques (using CO_2_ as solvent) such as supercritical fluid (SFE) and subcritical (SubCO_2_) extractions can improve their yields and quality in the extracts. SFE, SubCO_2_ and Soxhlet techniques were employed to enrich antioxidants in extracts from Sukari (SKSE), Ambara (AMSE), Majdool (MJSE) and Sagai (SGSE) date seeds. Extract yields were evaluated and modelled for SFE extract using response surface methodology. Significantly higher (*p* < 0.05) phenolics (143.48–274.98 mg GAE/100 g), flavonoids (78.35–141.78 mg QE/100 g), anthocyanins (0.39–1.00 mg/100 g), and carotenoid (1.42–1.91 mg BCE/100 g) contents were detected in extracts obtained using SFE and SubCO_2_ methods. The evaluation of in vitro antioxidant properties showed that SFE and SubCO_2_ seed extracts demonstrated promising antioxidant (13.42–23.83 µg AAE/mL), antiradical (228.76–109.69 µg/mL DPPH IC_50_), ferric reducing antioxidant power (1.43–2.10 mmol TE/100 g) and ABTS cation scavenging (375.74-717.45 µmol TE/100 g) properties that were significantly higher than Soxhlet extracts. Both SFE and SubCO_2_ techniques can be effectively utilized as innovative and environmentally friendly alternatives to obtain high quality antioxidant rich extracts from date seed. These extracts may have potential functional and nutraceutical applications.

## 1. Introduction

Date (*Phoenix dactylifera*) palm is an important plant in arid and semiarid regions of different Middle-Eastern and North African countries [1]. In Saudi Arabia alone approximately 1.5 million tonnes of date fruits were produced in 2019 with an estimated value of more than US$4 billion [2].

Date palm tree and fruit play important roles in the economic and social life of the people in the date producing countries [3]. Date by-products, such as date seed are rich in nutraceuticals and phytochemicals with different medicinal properties [4,5]. Different bioactive compounds have been detected in date fruit and seed, and their biological properties with potential industrial applications have been reviewed previously [6]. Huge quantities of date seed are produced during the manufacture of different products such as pitted dates, date powders, date syrup, date juice, and date confectionery. These date by-products have limited traditional uses; however, mostly they are wasted [7]. A recent study [1] demonstrated that there are important nutrients, including fatty acids (oleic, linoleic, lauric, palmitic, myristic and stearic acids being key fatty acids), tocopherols (predominant being α-tocotrienol) and phenolic acids (mainly gallic acid, syringic acid and catechins) in seeds of various date fruit varieties from Algeria, Morocco, Libya, Sudan and Pakistan. The contents of these nutrients are significantly reduced when date seeds are processed at higher temperature [8]. Development of functional foods and nutraceuticals require the extraction, isolation, identification, and quantification of bioactive compounds. Extraction, an important stage in this process, can be described by Fick’s second law of diffusion whereby the recovery of bioactive compounds from plant matrix is usually carried out using a solvent-based procedure [9]. The concentration of solvent, time, and temperature are important process parameters in these techniques, e.g., the Soxhlet method. However, these are energy intensive techniques, and they depend largely on concentrated toxic organic solvents, which may pose environmental concerns. Different innovative extraction techniques including ultrasound, supercritical fluid (SFE), microwave, and subcritical CO_2_-assisted (SubCO_2_) methods are being developed for the recovery of biologically valuable components from various plant matrices [9,10,11]. The supercritical state was first known back in 1822 and later on it was observed that supercritical fluids have promising solvating ability which may be dependent on the pressure. Currently, SFE is considered as an innovative and advanced technique to recover high quality bioactive compounds from various plant matrices [12]. Different types of solvents can be used as supercritical fluids; however, CO_2_, being odourless, colourless, non-flammable, nontoxic, inert, safe, inexpensive, recyclable and environmentally safe, is the most commonly used one. This solvent can assist the extraction of superior quality and heat-sensitive phytochemicals at lower temperatures in comparison to conventional methods [10]. SubCO_2_ method is an advanced technique that combines the advantages of both Soxhlet and CO_2_ extraction involving a continuous reformation of CO_2_ through boiling and condensation. The gas is circulated on its own in the system without the need for a pump, as required in case of the supercritical CO_2_ system. Moreover, the SubCO_2_ system works at low temperature (<31 °C), low pressure (<7.1 MPa), and requires lesser mechanical parts (unlike supercritical CO_2_) and, furthermore, residual solvent removal is not required [11]. The objectives of the current research work include isolation and recovery of bioactive compounds (phenolic, flavonoids, anthocyanins and carotenoids) from seeds of four different date varieties using conventional (Soxhlet) organic solvent-based and environment friendly SFE and SubCO_2_ (both using CO_2_ as extraction solvent) techniques. The yield of SFE date seed extracts was also optimized using response surface methodology. The biological properties of extracts obtained from date seed using these techniques were qualified and compared to find the optimal extract quality and recovery method.

## 2. Materials and Methods

### 2.1. Raw Materials and Sample Preparation

Four types of date fruits, namely Sukari, Ambara, Majdool and Sagai grown in Saudi Arabia in the year 2020 were purchased from the Seasonal Date Fruit Market, Riyadh, Saudi Arabia during October 2020. The date flesh and seed were separated manually. Seeds were dried in a vacuum oven at 50 °C for two weeks and ground using a universal cutting mill (Pulverisette 19, Fritsch, Idar-Oberstein, Germany). All chemicals used in the experiments were of analytical grade and obtained from Sigma-Aldrich (St. Louis, MO, USA).

### 2.2. Moisture Content Determination

The ground date seed samples were subjected to halogen moisture analyzer (Mettler Toledo-HB43) for moisture estimation using a Ca 2d–25 official method [13]. The first step was the fixation of the instrument at 105 °C prior to the placement of sample. A pre-dried 5 g sample of ground date seed was kept for 5 min in the sample handler (aluminium plate). The sample temperature was increased by halogen light in the instrument and results were recorded in percentage of weight on an automatic recorder. Triplicate measurements were carried out to determine the mean moisture content.

### 2.3. Extraction of Antioxidants from Date Seed

#### 2.3.1. Supercritical Fluid CO_2_ Extraction (SFE) for Date Seed

A unit of SFE consists of two pumps to pressurize CO_2_ and co-solvent separately. These pumps controlled the flow and pressure of respective solvents. Ethanol (30%) was used as co-solvent in the current study. CO_2_ was supplied in cylinders and the pump was connected with a coolant circulation system. The pump head was cooled by using ethylene glycol and deionized water mixture (50:50) that reduced the temperature down to –20 °C while circulating in the cooling jacket of the solvent pump. Extraction temperature (*X*_1_) was in the range of 35 °C to 70 °C, pressure (*X*_2_) was 15–40 MPa, and CO_2_ flow rate (*X*_3_) was kept 0.5–5 mL/min. Experiments were carried out using designed experiments [10,14,15] and a set of at least 18 trials were used for carrying out optimization experiments. Sample (20 g) of powdered date seed was kept in tightly sealed extraction vessel that was fixed in the column oven or extraction chamber. Solvent (CO_2_) was purged into the vessel and extraction was carried out at different condition of *X*_1_, *X*_2_ and *X*_3_. Once solutes were dissolved, the pressure at backpressure regulator became stable and extracts were collected there in a Schott bottle and dried using rotary vacuum evaporator. The extraction yield of the sample was calculated according to the following equation:

(1) $$Yield\;\left(\%\right)=\frac{Weight\;of\;extract}{Weight\;of\;ground\;sample}\times 100$$

All samples extract were placed in packages that prevented light penetration, sealed, and stored at −20 °C for various trials. Predictive model for the optimization of SFE for extract yield was based on the following equation:

(2) $$Y=\beta_{0}+\beta_{1}X_{1}+\beta_{2}X_{2}+\beta_{3}X_{3}+\beta_{11}X_{1}^{2}+\beta_{22}X_{2}^{2}+\beta_{33}X_{3}^{2}+\beta_{12}X_{1}X_{2}+\beta_{13}X_{1}X_{3}+\beta_{23}X_{2}X_{3}$$

where *Y* is the predicted response for the yield extracts (and can be denoted as *Y*_1_, *Y*_2_, *Y*_3_ and *Y*_4_ for seed extracts from Sukari, Ambra, Majdool and Sagai, respectively. *β*_0_ can be denoted as the offset term. The regression coefficients were *β*_2_ and *β*_3_ (linear); *β*_11_, *β*_22_ and *β*_33_ (quadratic) and *β*_12_, *β*_13_ and *β*_23_ (interaction). The *X*_1_, *X*_2_ and *X*_3_ are the independent variables referring to SFE temperature, pressure and CO_2_ flow rate, respectively. A similar process optimization approach using regression analyses and second-order empirical polynomial model was recently reported [16].

#### 2.3.2. Subcritical CO_2_ Extraction

The Subcritical CO_2_ extraction (SubCO_2_) of ground date seed was carried out following the method of previous studies [11,17] with some modifications. About 150 g of sample was treated with 75 mL pure ethanol (co-solvent) and kept in the extraction vessels. Once the system was set for extraction conditions (extractions cycles, heating and cooling temperatures), the liquid CO_2_ was made to flow into the vessel for the extraction of phytochemicals from date seeds. A 6 kg mass of CO_2_ was used in the SubCO_2_ system. The number of extraction cycles for each sample was 250 (approx. 12 h) at a pressure of 6.8 MPa and temperature of 29 °C. The extraction time also depended on the rates of re-boiling and condensation. The extract and depleted raw material recovery was carried out by depressurization of the system followed by evaporation of solvent in rotary evaporator. The total yield was determined in percentage of dried material used per run according to Equation (1). Extractions were carried out in triplicate and extracts were stored at −20 °C for further analyses.

#### 2.3.3. Soxhlet Extraction

About 25 g of ground sample was packed into Whatman^®^ cellulose extraction thimbles (Whatman PLC, Maidstone, UK) (28 × 100 mm) before placing in the Soxhlet extraction chamber. A total of 250 mL n-hexane was used as solvent for each 25 g sample in this extraction. The extraction was performed at around 70 °C for 16 h until the solution in the extraction chamber became clear. The extract was evaporated using a rotary vacuum evaporator under reduced pressure at 50 °C to remove the solvent. All of the crude extracts were stored in a refrigerator at −20 °C for further analysis. Extraction for each variety was repeated three times and the percentage of yield was calculated using Equation (1).

### 2.4. Analysis of Antioxidants in Date Seed Extracts

#### 2.4.1. Determination of Total Phenolic Contents

A modified method using Folin Ciocalteu (FC) reagent [18] was employed for the estimation of total phenolic compounds (TPC). A 200 μL extract sample, obtained by mixing 10 mg extract in 100 mL methanol, or a standard solution with variable concentrations was dissolved in 400 μL FC reagent and diluted to 4.6 mL with the addition of 4 mL of deionized water. After 10 min incubation at room temperature, a 10% sodium carbonate (1 mL) solution was added and mixed thoroughly. Then the test mixture was kept at room temperature for 2 h followed by absorbance values’ measurement at 765 nm using a spectrophotometer. In order to obtain a standard curve, methanolic solutions (samples) of gallic acid were prepared in variable concentrations. The results from extract samples were compared with those of standard curves to express TPC of samples as mg gallic acid equivalent (GAE) per 100 g. Each sample was analysed in triplicate.

#### 2.4.2. Determination of Total Flavonoids Contents

A colorimetric procedure was used for assessment of total flavonoids in date seed extract, as explained by Biglari et al. [19]. Extracts were dissolved in methanol and then 1 mL of this liquid was mixed with 0.3 mL each of sodium nitrite (5%) and aluminium chloride (10%) solution followed by 5 min incubation at room temperature. Afterwards, a 2 mL sodium hydroxide (1 M) was dissolved, and total volume of the reaction mixture increased to 10 mL with the addition of distilled water. A pink colour developed in this mixture and prior to absorbance (510 nm) the contents were mixed again. Quercetin was used to obtain a calibration curve. The quantities of total flavonoids were expressed as mg of quercetin equivalent (QE) per 100 g (mgQE/100 g) of extract. Each sample was analysed in triplicate.

#### 2.4.3. Total Anthocyanins Evaluation

The analysis of total anthocyanins in date seed and flesh extract was based on previous method [20]. The nutraceutical sample was mixed in methanol as explained in total phenolic determination and 1 mL of this liquid was mixed with 10 mL ethanol (50%) followed by 10 min centrifugation at 1800× *g*. A sample of 200 μL was taken from the supernatant and mixed with 3.8 mL of HCl (1 M) followed by 3 h incubation at room temperature. The values for the absorbance (A) of the resultant mixture were recorded, after mixing again, at 520 nm using HCl as a blank. A 1% solution of malvidin-3-glucoside was prepared in the same manner as explained earlier, and then absorbance (B) was recorded. Each experiment was performed in triplicate. The calculation of total anthocyanins was based on the following formula and analysis of each sample was replicated thrice.

$$Total\;anthocyanins\;\left(mg\;g^{-1}\right)=\frac{A\times dilution\;factor\times 1000}{B}$$

#### 2.4.4. Determination of Total Carotenoids

Total carotenoids quantification was based on Ranjith et al.’s [21] method by using β-carotene as a standard. A sample of 1 g of the extract was mixed with 0.5 mL of NaCl (5%) solution in water by 30 s vortex mixing and 10 min centrifugation at 3000× *g*. The resultant supernatant was diluted further with n hexane and absorbance values were recorded at 460 nm. β-carotene solutions of varying concentrations were used to obtain a standard curve, and total carotenoid in date fruit nutraceuticals was expressed as mg of β-carotene equivalent (BCE) per 100 g of the sample.

### 2.5. Determination of Antioxidant Activities of Date Seed Extracts

#### 2.5.1. Antioxidant Activity Determination Using Phosphomolybdenum Complex

Antioxidant activity determination by phosphomolybdenum complex method followed the method developed by Prieto et al. [22]. Briefly, 100 µg of tested sample was dissolved in 1 mL of methanol and 400 µL of this solution was mixed with 4 mL of a reagent solution (0.6 M sulphuric acid, 2 mM sodium phosphate and 4 mM ammonium molybdate). Another solution was prepared by mixing 4 mL of reagent solution and 1 mL of methanol and used as a blank. Samples were kept in caped and sealed test tubes incubated at 95 °C for 1 h using a water bath. Once the reaction was completed, contents cooled down using running tab water and measurement of absorbance at 695 nm was accomplished against a blank. The results were expressed as relative to those of ascorbic acid, which was used in preparation of the calibration curve following the above procedure in the absence of extract samples. Each experiment was repeated in triplicate.

#### 2.5.2. Free Radical Scavenging Activity (DPPH Method)

A commonly used 1, 1-diphenyl-2-picrylhydrazyl (DPPH) method [23] was used to assess radical scavenging properties of date seed neutraceuticals obtained by SFE methods. In this method, the dark blue coloured DPPH solution was reacted with bioactive compounds, which resulted in a change of colour to a lighter shade. A 1 mL extract solution (100 μg extract/mL methanol) was reacted with 2 mL DPPH solution obtained by mixing 1 mg of DPPH in 100 mL methanol. Followed by vigorous mixing, the mixture was allowed to stand for 5 min at room temperature and spectrophotometer readings were taken at 517 nm. The lower the IC_50_ value of absorbance, the higher the radical scavenging ability of the extract. This was expressed as percentage of radical scavenged. Each experiment was repeated in triplicate.

#### 2.5.3. Ferric Reducing Antioxidant Power (FRAP) Assay

The FRAP assay was based on a method of Benzie and Szeto [24] after some modifications. A FRAP solution was prepared by using 300 mM acetate buffer (pH 3.6), ferric chloride hexahydrate (20 mM in water), and TPTZ (10 mM in 40 mM HCl solution). The nutraceutical rich date fruit extracts were dissolved in 3 mL of FRAP solution to maintain their concentrations in the range of 0.02–1 mg/mL followed by 30 min incubation at room temperature. A coloured product due to ferrous tripyridyl triazine complex was formed the absorbance values of which were recorded at 593 nm. A calibration curve was also formed using trolox (6-hydroxy-2,5,7,8-tetramethylchroman-2-carboxylic acid), and FRAP activity was expressed as mMTE/100 g. Each experiment was repeated in triplicate.

#### 2.5.4. ABTS Cation Radical-Scavenging Assay

ABTS was abbreviated form of 2, 2-azinobis, 3-ethylbenzothiazoline, 6-sulfonic acid diammonium salt. The cation radical scavenging activity was based on the method of Re [25]. The preparation of a stock solution involved reaction of ABTS (7 mM) with potassium persulfate (2.45 mM) and by dark incubation at room temperature for 12–16 h for the generation of ABTS cation chromophore. Absolute ethanol was used to dilute this mixture in order to achieve a spectrophotometer absorbance of 0.700 ± 0.01 at 734 nm. Extracts (10–100 µg/mL ethanol) aliquot was mixed with 3 mL of ABTS reagent followed by incubation at 23 °C. The absorbance values were recorded after 6 min, and each experiment was repeated in triplicate. Trolox was used for preparing the calibration curve, and the result was presented as μmol TE/100 g.

### 2.6. Statistical Analyses

The extraction studies and analytical measurements were carried out in triplicate and the experimental results obtained were expressed as means ± SD. A statistical analysis system (SAS, version 9.4, SAS Institute, Cary, NC, USA) software was used for data evaluation using regression analysis and analysis of variance techniques. Significance was defined as *p* < 0.05.

## 3. Results and Discussion

### 3.1. Supercritical Fluid Extraction and Response Surface Optimization

**Figure 1:**
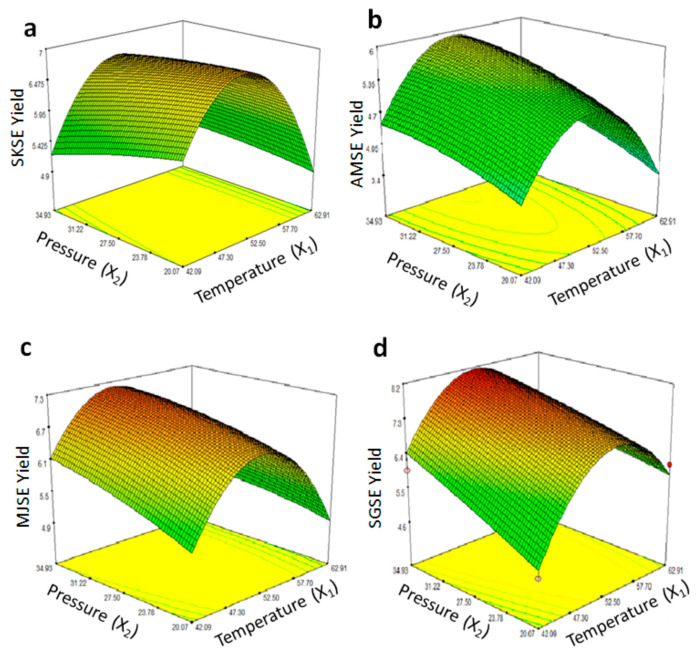
Response surface plots of process variables (temperature and pressure) and extract yields from Sukari (SKSE, (**a**)), Ambara (AMSE, (**b**)), Majdool (MJSE, (**c**)) and Sagai (SGSE (**d**)) seed extracts obtained using supercritical fluid (CO_2_) extraction (SFE).

The moisture contents of ground seed samples used for extraction of nutraceuticals were 8.90 ± 0.74, 6.96 ± 0.65, 6.96 ± 0.91, and 7.79 ± 0.66%, from Sukari, Ambara, Majdool and Sagai dates, respectively. The yields of date seed extracts (obtained by SFE) are shown in Table 1. The regression analysis of data obtained for Sukari date seed extract (SKSE) showed that it was significantly (*p* < 0.05) affected by the linear and quadratic terms of the model shown in Equation (2). The relationship of the SSE yield and that of SFE temperature and pressure is depicted in Figure 1a and with R2 value of 0.9489. We may increase extract yield by increasing temperature or pressure, at a fixed CO_2_ flow rate. A predictive model for SKSE using SFE can be presented using coefficients of significant terms as follows:

(3) $$Y_{SKSE}=-32.275710+1.408322X_{1}+0.052476X_{2}-0.013584X_{1}^{2}-0.000994X_{2}^{2}$$

where *Y* is the extract yield, *X*_1_ is the temperature and *X*_2_ pressure during SFE. The total model had a significant value (*p* = 0.0003).

The regression analysis of the extract yields obtained from Ambara date seed (AMSE) also showed significant effects of the main variables (*X*_1_ and *X*_2_) and this revealed that the process was significantly affected by linear and quadratic terms of these variables. A predictive mode with *p* value of 0.0029 is presented as follows:

(4) $$Y_{AMSE}=-35.279577+1.408665X_{1}+0.155612X_{2}-0.013846X_{1}^{2}-0.002625X_{2}^{2}$$

The response surface plots showing the relationship between significant process variables is shown in Figure 1b. The modelling of SFE process for Majdool date seed extract (MJSE) showed similar significant effects of linear and quadratic process variables, and a predictive model with *p* < 0.001 can be presented, based on regression analysis results, as follows:

(5) $$Y_{MJSE}=-36.738563+1.492322X_{1}+0.143659X_{2}-0.013577X_{1}^{2}-0.001683X_{2}^{2}$$

The regression analysis results are presented as a response surface plot for the SFE of Majdool seed extract, as shown in Figure 1c. In case of Sagai date seed extract (SGSE), the predictive model for response surface optimization (Figure 1c) can be presented by using values of significant coefficients as follows:

(6) $$Y_{SGSE}=-41.446689+1.749680X_{1}+0.153705X_{2}-0.015749X_{1}^{2}-0.000787X_{2}^{2}$$

Process optimization can play a key role in biochemical processes; e.g., the extraction of nutraceuticals and hence and enhanced extract yield may correlate significantly with the recovery of bioactive compounds. In order to achieve this objective, different statistical approaches may be employed [15]. In case of the current study, the maximal extract yields from SFE were 8.13, 7.52, 7.72 and 8.28% in case of SKSE, AMSE, MJSE and SGSE and they were obtained at temperature of 52.50 °C, pressure of 27.50 MPa and CO_2_ flow rate of 5 mL/min (Table 1 and Table 2). The yields for extracts prepared during 18 experimental runs were compared to find out the optimal yield. A predictive modelling (Equations (3)–(6)) approach was also adopted for the date seed types. Subsequent experiments can also be carried out to predict optimum variables for each date seed type, and validation experiments may also be carried out. A similar study [26] reported optimization of oil yield from Algerian date seed, and SFE pressure, temperature, and particle size of the date seed powdered samples were selected as independent process variables. The study also included characterization of fatty acids in recovered oil. The oil yields were comparable with those obtained using Soxhlet system. A solvent (n-hexane and chloroform) based procedure was also recently reported to recover oil from Sukkari date seed [27] and process variables such as grain size, drying method, solvent type, and feed to solvent ratio were optimized. The current study differs from these reported ones as oil was not recovered and the main objective was to enhance the extract yield and, later, the contents of bioactive compounds in the extract. Generally, a correlation between extract yield and bioactive compounds contents is often reported [28]. These high extract yields samples were used for further analysis and comparison with other extraction methodologies.

### 3.2. Soxhlet and Subcritical CO_2_ Extraction Yields

The results of Soxhlet and Subcritical-CO_2_ extraction (SubCO_2_) yields from seeds of four different date varieties are shown in Table 2 and results were compared with those from SFE. The Soxhlet and SubCO_2_ experimental conditions were based on previous studies [11,16] and preliminary trials. It can be observed that both SubCO_2_ and SFE were comparable in terms of extract yields, and produced significantly (*p* < 0.05) higher extract yields in comparison to the Soxhlet systems. In SubCO_2_ and SFE systems, the varieties do not seem to have significant effects on yields; however in the case of the Soxhlet system, the highest (5.66%) yield was observed for MJSE whereas the lowest (3.44%) was observed in case of Sagai seed. Both SubCO_2_ and SFE resulted in significant improvement of the recovery of extracts from all the varieties. The SubCO_2_, which is a modified or advanced form of Soxhlet extraction involving use of liquid CO_2_ and pure ethanol as co-solvent (in current study) thus significantly enhanced the solubility strength of CO_2_ at a lower temperature of 29 °C [11,16]. Furthermore, n-hexane, which was used during the Soxhlet extraction process may cause environmental and health problems, and maintenance of higher extraction temperature for a prolong period may also be energy intensive [29]. It is generally understood that the extraction yields are mainly dependent on the extraction temperature [9,20] and, conforming to this, the yields were slightly higher in Soxhlet (70 °C) in comparison to SubCO_2_ (29 °C) and SFE (29 °C). Hence, in the current study, extraction pressure (as used in SubCO_2_ and SFE systems) seems to play a significant role in enhancing the extraction yields from date seed. This advantageous effect of high pressure may be due to the increase of solvent density and solvation power of CO_2_, and similar effects of using high pressure extraction methods such as SFE were observed on the global yield of extract from feijoa leaf in comparison to low pressure or conventional high temperature methods (Soxhlet) [30]. The Soxhlet system was dependent on the use of toxic n-hexane, whereas the other two systems used ethanol as co-solvent. A co-solvent is generally an organic solvent (30% ethanol for SFE and 99.9% ethanol for SubCO_2_) added to the main solvent (CO_2_ in SubCO_2_ and SFE) to modify or improve the solubility properties of CO_2_ against polar compounds [11]. Ethanol is preferable due to its abundance, non-toxicity (food grade and pharmacopoeia grade), purity, and environmental friendliness [9,15].

### 3.3. Antioxidants and Date Seed Extracts Obtained Using Different Techniques

The total phenolic (TPC), total flavonoids (TFC), total anthocyanins (TAC) and total carotenoids contents (TCC) of extracts obtained from seeds of four different date fruit verities using soxhlet, SubCO_2_, SFE methods are presented in Table 3.

#### 3.3.1. Phenolic and Flavonoid Compounds

Phenolic compounds are universally distributed phytochemicals present in different types of plant matrices that include fruits and vegetables. These are considered secondary metabolites produced through the shikimic acid and phenylpropanoid pathways. Although they are not considered as nutrients, phenolic compounds are associated with various health-related benefits [31]. The TPC of date seed extracts obtained using different extraction methods were significantly (*p* < 0.05) different among different varieties and similar significant effects of methods on TPC were also observed. The extracts obtained using SubCO_2_ method had higher TPC than those from Soxhlet and SFE methods. The highest (274.98 mg GAE/100 g) and the lowest (99.13 mg GAE/100 g) TPC values were detected in SubCO_2_-MJSE and Soxhlet-MJSE extracts revealing that how significant an extraction method can be in the recovery of TPC from a similar plant tissue, Majdool date seed in the current study. The TPC contents were the highest in SubCO_2_ extracts (227.93–274.98 mgGAE/100) followed by SFE extracts (143.48–186.44 mgGAE/100 g) from date seed.

TPC were observed as the most abundant group, followed by total flavonoids (TFC) in date seed extracts. Flavonoids are a group of polyphenolic compounds often found in flowers, leaves and seeds of different plants as secondary metabolites. They are generally present as glycosides in plant cell vacuoles and based on their structure categorized into seven different subclasses, namely flavonols, flavones, isoflavones, anthocyanidins, flavanones, flavanols, and chalcones. These polyphenols are gaining increasing attention due to their biological properties [32]. Similar to the results for TPC, SubCO_2_ extraction seems to have profound effects on the content of TFC as all top four yields for these types of compounds were detected in date seed extracts obtained using this method. The highest TFC were detected in SubCO_2_-SGSE (141.78 mgQE/100 g) followed by SubCO_2_-MJSE (123.23 mg QE/100 g). The TFC of SFE and Soxhlet extracts ranged between 74.92–90.04 and 47.30–60.43 mgQE/100 g, respectively. It is observed that SubCO_2_ technique can effectively improve the recovery of TPC and TFC from date seeds. Further process modifications, such as use of subcritical water extraction (SCWE) technique, may further enhance the contents of TPC and TFC from date seed, as observed by Li et al. [33]. A recent study reported the use of SCWE to recover phenolic compounds and sugars from microwave-pretreated date fruit pulp (MW-FP), and promising results were obtained when compared to conventional hot water extraction [34]. It was reported [33] that use of high extraction temperature (150 °C), 20 min extraction time and optimal feed mixture concentration (6% by weight) can increase TPC and TFC recovery (from Hallawi date seed) up to 1060 mgGAE/100 g and 414 mg QE/100 g, respectively. However, it is important to know that SubCO_2_ and SFE techniques, used in the current study, involved an extraction temperature of 29 and 52.5 °C, respectively. These methods are less energy-extensive and considered green methods and believed to improve the quality and functional properties of the recovered temperature sensitive phytochemicals [35,36,37].

#### 3.3.2. Carotenoids and Total Anthocyanins

Extracts from seeds of four different date varieties obtained using three different techniques were also analysed for their total anthocyanin (TAC) and carotenoids contents (TCC), and the results are presented in Table 3. The TAC contents of different types of extracts ranged between 0.24–1.00 mg/100 g. In general, the Soxhlet extracts showed the lower TAC values, whereas those of SubCO_2_ extracts were higher. Significant (*p* < 0.05) differences in TAC and TCC were observed depending on the variety and the extraction method employed. The SFE method seemed to be more effective in the recovery of TCC from date seed. The highest TCC (1.91 mgBCE/100 g) was noted in SFE-SGSE and the lowest (0.94 mgBCE/100 g) in Soxhlet-SGSE, showing that selection of appropriate methods can significantly improve the recovery of carotenoids from the seed of the same date variety such as Sagai.

Anthocyanins are coloured compounds and are a sub-class of dietary flavonoids. Their colour generally depends on the pH of fruit, molecular structure, and glycoside substituents on the anthocyanidin core. Studies involving in vitro experiment and some animal trials show their valuable biological properties such as antioxidant, anti-inflammatory, antimicrobial, and anti- carcinogenic activities [38,39]. Carotenoids are found in different plants, fruits, and vegetables as another type of plant pigments and different reported health benefits [40]. Al-Farsi et al. (2005) [41] evaluated the phytochemical contents in fresh and sun-dried dates of three native varieties (Fard, Khasab, and Khalas) and observed the TAC and TCC in the ranges of 0.24−1.52 mg/100 g and 1.31−3.03 mg/100 g, respectively. Another study [42] in which seed oil from different date varieties were used for the preparation of extract reported the TCC as 17.57–12.350.03 mg/kg oil, TPC as 58.04–181.03 mgGAE/100 g and TFC ranging 22.81 to 53.41 mg RE/100 g oil. These reported values demonstrate certain agreements (in certain cases, much lower antioxidant contents) with those reported in the present study, approving the effectiveness of using innovative extraction methodologies and optimization techniques for enhancing the recovery of different types of antioxidants from date seed.

### 3.4. Antioxidant Properties of Date Seed Extracts

The estimation of in vitro biological or antioxidant properties is the most common approach for determining the functional properties of phytochemicals. Different degenerative processes in the human body are correlated with presence and/or generation of free radicals, which in turn enhance harmful oxidative processes. Different phytochemicals such as phenolics, flavonoids, anthocyanins, carotenoids, certain vitamins, enzymes, and fatty acids are capable of exerting prophylactic and curative effects on these free radicals [43]. The antioxidant properties of extracts (containing phytochemcials) from the seed of four different date varieties obtained using three different techniques (soxhlet, SubCO_2_, SFE) and as determined using different in vitro methods such as phosphomolybdenum complex (antioxidant), free radical scavenging activity (DPPH), ferric reducing antioxidant power (FRAP), and ABTS cation radical-scavenging (ABTS) assays are shown in Table 4. All these methods involved the use of colorimetric measurements of absorbance values of the products in the reaction mixtures. Some of these tests include formation of blue-coloured end products in antioxidant evaluation using phosphomolybdenum complex and the reactions of antioxidants in the extract with free DPPH radicals, ferric complex, and ABTS cations [43].

#### 3.4.1. Antioxidant Property

The antioxidant activity as measured by using phosphomolybdenum complex method is reported in Table 4 and it can be observed that the activity varied significantly (*p* < 0.05) with the extraction method. For instance, we can note that the highest antioxidant (23.83 µgAAE/mL) was observed in SubCO_2_-MJSE followed by SFE-MJSE (22.62 µgAAE/mL) and SX-MJSE (21.12 µgAAE/mL), showing that Majdool seed had better overall activity when measured using this method, no matter which extraction technique was used. The lowest (9.29 µgAAE/mL) in the SX-AMSE and Ambara seed generally showed lower activity in the extract prepared using all three techniques, thus demonstrating the effects of variety on the antioxidant potential of the date seed. These results showed better antioxidant potential of date seed than those reported by Ourradi et al. [42] who used hexane and methanol aqueous solution to obtain extract from date seed oil.

#### 3.4.2. Free Radical Scavenging Activity

The DPPH radical scavenging activity as demonstrated by IC_50_ values (i.e., the concentration of antioxidants in the extract required to reduce the initial DPPH concentration by 50%) of various date seed extracts, where a lower value indicates higher radical scavenging ability [44] is presented in Table 4. Consistent with antioxidant acidity results reported in the previous section, the lowest (109.69 µg/mL) IC_50_ value was observed in SubCO_2_-MJSE demonstrating this extract’s strong potential to scavenge free DPPH radicals. The highest IC_50_ value (353.83 µg/mL) or the weakest potential against DPPH free radical was observed in SX-SGSE, which was significantly improved in SGSE when prepared using advanced techniques demonstrating a significant (*p* < 0.05) effect of the recovery method. In terms of DPPH IC_50_ value, SubCO_2_ date seed extracts demonstrated the best potential followed by those from SFE, where this value ranged from 110–229 µg/mL whereas in case of Soxhlet method the value remains close or higher than 300 µg/mL. In a previous study [45], the DPPH radical scavenging activity of date seed extracts from different date varieties were reported to be 78–80%, showing that this by-product can be effectively used for health-product formulations and nutraceuticals. The current study reports the effectiveness of using green and innovative methods for enhancing the health-benefiting effects of date seed antioxidants. In another study [42], the antiradical activity of extracts from date seed oil were reported to range between 17.28 and 47.37%; however, in this study, only conventional heating methods, centrifugation and Soxhlet methods were employed to obtain date seed oil and extracts.

#### 3.4.3. Ferric Reducing Antioxidant Power

The FRAP test is a typical single electron transfer assay involving measurement of reduction reaction of ferric ions (Fe^3+^)-ligand to ferrous ions (Fe^2+^) that results in the formation of a blue-coloured complex after reaction with antioxidants under acidic conditions. The main linking ligand for irons ions used in this test is tripyridyltriazine (TPTZ) [43]. Similar to previous in vitro antioxidant assays, FRAP values of different seed extracts (Table 4) also revealed that the SubCO_2_ extraction method resulted in the extraction of phytochemicals with significantly (*p* < 0.05) higher FRAP values, with SubCO_2_-SKSE topping the list (2.10 mmTE/100 g) followed by MJSE (1.99 mmTE/100 g) and SGSE (1.91 mmTE/100 g) obtained using the same extraction method. The application of SFE improved the FRAP value of SGSE, which was 1.85 mmTE/100 g, and all extracts obtained using SFE method showed significantly (*p* < 0.05) higher FRAP values in comparison to the Soxhlet method. The FRAP values for AMSE were lower than those of other date seed extracts when compared within each extraction technique. Hence, certain varietal influence on FRAP values of date seed can also be observed along with evident effects of the extraction techniques, which is consistent with previous reports [1,45].

#### 3.4.4. ABTS Cation Radical Scavenging

This is one of the in vitro antioxidant assays (the other one being DPPH antiradical activity) which can be regarded as a mixed assay involving transfer of an electron and hydrogen atom from the main reagent. An organic cation radical is generated from 2,2ʹ-Azinobis-(3-ethylbenzothiazoline-6-sulfonic acid (ABTS) which is oxidized by the antioxidants present in the natural extract, date seed extract in the current study [43,46,47]. The results presented in Table 4 demonstrated significant (*p* < 0.05) effects of both the date seed variety and extraction techniques on the ABTS cation radical scavenging activity of date seed extracts from four different Saudi date varieties and prepared using Soxhlet, SFE, and SubCO_2_ methods. Based on the results, a particular date variety cannot be selected for generally higher ABTS activity; however, among extraction techniques, SubCO_2_ proved to show higher activities for all four types of date seed extracts. The highest activity was monitored in SubCO_2_-MJSE (717.45 µmolTE/100 g) followed by SubCO_2_-AMSE (648.33 µmolTE/100 g) and SubCO_2_-SKSE (628.45 µmolTE/100 g). The ABTS cation scavenging activity of SFE extracts of four different date seeds remained in the range of 375–444 µmolTE/100 g whereas that of Soxhlet extracts ranged between 271–307 µmolTE/100 g. Ourradi et al. [42] found the ABTS activity of extracts from date seed oil from eight Moroccan dates ranging from 3.10 to 8.83 mg AAE/100 g. The date seed oil [42] was prepared using petroleum ether in a Soxhlet system followed by another extraction treatment using hexane, methanol, and centrifugation. The TPC and antioxidant activity (ABTS radical scavenging) observed in the extracts from date fruit using SCWE were 1.26 g/100 MW-FP 92%, respectively [34].

## 4. Conclusions

Date fruit by-products such as seed can be a valuable source of antioxidants for nutraceuticals, and functional food applications. The utilization of innovative, environmentally friendly, and optimized techniques can significantly improve the recovery and quality of the extracts. The extracts can be valuable sources of bioactive compounds having possible health benefits. In the present study, two innovative and low temperature antioxidant recovery methods, namely SFE and SubCO_2,_ were employed and compared for the yield, antioxidant contents, and antioxidant properties of extracts from seed of four different date varieties with conventional Soxhlet technique. Optimization and predictive modelling of SFE method using response surface technique were also carried out for each date seed type. Yields of both SFE and SubCO_2_ extracts were significantly higher than in the Soxhlet method (recovery percentage <6% and using 70 °C and n-hexane for 16 h process). Although the extract yield may be important from a processor’s viewpoint, the contents of individual functional compounds may be more crucial in determining the health potential and quality of obtained extracts. The extracts prepared using SFE and SubCO_2_ methods were rich in antioxidant (phenolic, flavonoid, anthocyanin, carotenoid) compounds and their contents were significantly higher than in the extracts obtained using conventional methods. The antioxidant properties of these extracts were also evaluated using phosphomolybdenum complex, DPPH, FRAP and ABTS methods. The extracts obtained using innovative methods (SFE and SubCO_2_ methods), employing low temperature and CO_2_ as extraction solvent/medium, exhibited significantly higher antioxidant properties than the conventionally prepared ones. Among most of the antioxidant related parameters evaluated for all the extracts, SubCO_2_ extracts showed maximal values. These properties seem to be variably dependent on the date seed variety as well. Hence, it may be inferred that an innovative approach for extract preparation from fruit and plant by-products such as date seed can improve their functional quality and antioxidant contents. Further studies can also be carried out for in vivo evaluation of date seed antioxidant and their application in functional foods and nutraceuticals. Chromatographic separation and quantification of extract obtained using these techniques may help to understand the impact of each recovery method on the contents of individual bioactive compounds.

## Figures and Tables

**Table 1:** Supercritical fluid extraction (SFE) yields from date seeds using designed experiments.

Run	Process Variables of SFE			Response Variable (Yield %)			
	Temperature °C (*X*_1_)	Pressure MPa (*X*_2_)	CO_2_ Flow mL/min (*X*_3_)	Sukari Seed (*Y*_1_)	Ambara Seed (*Y*_2_)	Majdool Seed (*Y*_3_)	Sagai Seed (*Y*_4_)
1	62.91	20.07	4.09	5.23 ± 0.26	4.62 ± 0.13	5.26 ± 0.45	6.13 ± 1.41
2	62.91	34.93	4.09	4.89 ± 0.18	4.78 ± 0.32	5.19 ± 0.62	6.33 ± 1.22
3	52.50	27.50	2.75	5.91 ± 0.33	6.95 ± 0.24	6.61 ± 0.85	6.85 ± 0.98
4	52.50	40.00	2.75	6.45 ± 0.25	6.76 ± 0.17	6.85 ± 1.01	7.73 ± 1.22
5	52.50	27.50	2.75	5.94 ± 0.13	6.12 ± 0.42	6.48 ± 0.96	7.49 ± 1.45
6	52.50	27.50	2.75	6.11 ± 0.75	6.30 ± 0.52	6.37 ± 0.49	7.27 ± 1.05
7	52.50	27.50	0.50	5.54 ± 0.11	4.32 ± 0.17	5.10 ± 0.54	5.75 ± 0.72
8	62.91	20.07	1.41	4.72 ± 0.08	3.22 ± 0.09	4.54 ± 0.41	5.82 ± 0.65
9	52.50	27.50	2.75	6.69 ± 0.25	4.81 ± 0.17	5.92 ± 0.65	6.99 ± 1.43
10	62.91	34.93	1.41	5.24 ± 0.18	4.07 ± 0.15	4.21 ± 0.37	4.83 ± 0.62
11	70.00	27.50	2.75	1.74 ± 0.06	1.60 ± 0.06	1.90 ± 0.02	2.18 ± 0.04
12	52.50	15.00	2.75	5.53 ± 0.41	4.71 ± 0.16	5.29 ± 0.47	6.25 ± 0.79
13	42.09	20.07	1.41	4.44 ± 0.22	3.82 ± 0.08	4.14 ± 0.54	4.73 ± 0.24
14	42.09	34.93	4.09	4.92 ± 0.13	4.97 ± 0.34	5.98 ± 0.76	5.98 ± 0.69
15	42.09	20.07	4.09	5.87 ± 0.35	4.70 ± 0.33	5.33 ± 0.64	4.64 ± 0.29
16	42.09	34.93	1.41	3.81 ± 0.10	3.92 ± 0.21	3.87 ± 0.11	4.63 ± 0.35
17	35.00	27.50	2.75	2.23 ± 0.08	2.21 ± 0.02	2.45 ± 0.04	2.4 ± 0.18
18	52.50	27.50	5.00	8.13 ± 1.04	7.52 ± 0.85	7.72 ± 1.17	8.28 ± 0.97

**Table 2:** The percentage yields of date seed samples using Soxhlet and Subcritical CO_2_ (SubCO_2_) and supercritical fluid (SFE) extraction techniques.

Sample	Percentage Yields		
	Soxhlet (70 °C for 16 h Using n-hexane)	SubCO_2_ (250 cycles, 29 °C, 6.8 MPa, 12 h, Ethanol as Co-Solvent)	SFE (52.5 °C, 27.50 MPa, 5 mL CO_2_/min)
Sukari seed	5.21 ± 0.31 ^aB^	7.86 ± 0.59 ^aA^	8.13 ± 1.04 ^aA^
Ambara seed	4.31 ± 0.29 ^abB^	8.56 ± 0.58 ^aA^	7.52 ± 0.85 ^aA^
Majdool seed	5.66 ± 0.56 ^aB^	7.51 ± 0.86 ^aA^	7.72 ± 1.17 ^aA^
Sagai seed	3.44 ± 0.19 ^bB^	7.78 ± 0.61 ^aA^	8.28 ± 0.97 ^aA^

Data is represented by means ± standard deviations. Means with small letter superscripts are significantly different (*p* < 0.05) within columns and those with capital letter superscripts are significantly different within rows.

**Table 3:** Phytochemicals and total antioxidant compounds of date seed extracts obtained by using different extraction methods.

Extraction Methods	Extract Samples	TPC (mgGAE/100 g)	TFC (mgQE/100 g)	TAC (mg/100 g)	TCC (mgBCE/100 g)
Soxhlet	Sukari seed	124.27 ± 3.48 ^j^	60.43 ± 0.83 ^i^	0.30 ± 0.02 ^g^	1.04 ± 0.05 ^f^
	Ambara seed	114.01 ± 2.02 ^k^	53.08 ± 0.38 ^j^	0.29 ± 0.02 ^g^	0.96 ± 0.02 ^fg^
	Majdool seed	99.13 ± 1.68 ^l^	47.30 ± 0.63 ^k^	0.36 ± 0.02 ^f^	1.18 ± 0.03 ^e^
	Sagai seed	125.16 ± 2.18 ^j^	54.36 ± 0.87 ^j^	0.24 ± 0.02 ^h^	0.94 ± 0.04 ^g^
SubCO_2_	Sukari seed	263.09 ± 2.19 ^b^	98.01 ± 0.91 ^d^	0.60 ± 0.02 ^c^	1.46 ± 0.03 ^d^
	Ambara seed	254.52 ± 1.60 ^c^	106.64 ± 1.06 ^c^	0.80 ± 0.02 ^b^	1.62 ± 0.03 ^c^
	Majdool seed	274.98 ± 1.59 ^a^	123.23 ± 2.45 ^b^	0.78 ± 0.01 ^b^	1.71 ± 0.03 ^b^
	Sagai seed	227.93 ± 2.52 ^e^	141.78 ± 2.59 ^a^	1.00 ± 0.02 ^a^	1.91 ± 0.04 ^a^
SFE	Sukari seed	156.43 ± 1.86 ^h^	78.35 ± 1.02 ^g^	0.39 ± 0.02 ^f^	1.59 ± 0.03 ^c^
	Ambara seed	143.48 ± 2.12 ^i^	85.96 ± 1.00 ^f^	0.45 ± 0.02 ^e^	1.42 ± 0.03 ^d^
	Majdool seed	171.44 ± 1.40 ^g^	90.04 ± 0.63 ^e^	0.51 ± 0.02 ^d^	1.73 ± 0.03 ^b^
	Sagai seed	186.44 ± 0.89 ^f^	74.92 ± 0.90 ^h^	0.46 ± 0.01 ^e^	1.91 ± 0.03 ^a^

Data is represented by means ±standard deviations. SubCO_2_: Subcritical CO_2_ extraction; SFE: supercritical fluid extraction; TPC: Total phenolic contents; TFC; Total flavonoids contents; TAC: total anthocyanins contents and TCC: total carotenoids contents; Means with small letter superscripts are significantly different (*p* < 0.05) within columns.

**Table 4:** Antioxidant/biological properties of date seed extracts obtained by using different extraction methods.

Extraction Methods	Extract Samples	Antioxidant (µg AAE/mL)	DPPH IC_50_ (µg/mL)	FRAP (mmolTE/100 g)	ABTS (μmol TE/100 g)
Soxhlet	Sukari seed	11.81 ± 0.70 ^g^	337.79 ± 4.26 ^b^	1.27 ± 0.03 ^j^	271.59 ± 3.21 ^i^
	Ambara seed	9.29 ± 0.25 ^h^	317.45 ± 1.46 ^c^	0.97 ± 0.03 ^k^	271.96 ± 12.67 ^i^
	Majdool seed	21.12 ± 0.50 ^c^	297.71 ± 3.55 ^d^	1.34 ± 0.02 ^i^	303.60 ± 4.88 ^h^
	Sagai seed	16.03 ± 0.45 ^e^	353.83 ± 1.92 ^a^	1.39 ± 0.02 ^hi^	306.29 ± 4.67 ^h^
SubCO_2_	Sukari seed	16.14 ± 0.91 ^e^	162.15 ± 1.97 ^i^	2.10 ± 0.05 ^a^	628.45 ± 9.97 ^c^
	Ambara seed	13.52 ± 1.35 ^f^	156.27 ± 1.68 ^j^	1.58 ± 0.04 ^f^	648.33 ± 10.08 ^b^
	Majdool seed	23.83 ± 1.51 ^a^	109.69 ± 1.47 ^l^	1.99 ± 0.05 ^b^	717.45 ± 10.62 ^a^
	Sagai seed	18.79 ± 1.40 ^d^	121.42 ± 1.90 ^k^	1.91 ± 0.02 ^c^	545.70 ± 10.87 ^d^
SFE	Sukari seed	13.87 ± 0.55 ^f^	228.76 ± 4.67 ^e^	1.55 ± 0.02 ^g^	375.74 ± 4.75 ^g^
	Ambara seed	13.42 ± 1.01 ^f^	207.76 ± 1.77 ^f^	1.43 ± 0.02 ^h^	410.71 ± 4.91 ^f^
	Majdool seed	22.62 ± 0.71 ^bc^	190.04 ± 0.21 ^h^	1.68 ± 0.03 ^e^	424.84 ± 13.15 ^ef^
	Sagai seed	18.12 ± 1.21 ^d^	198.39 ± 1.61 ^g^	1.85 ± 0.03 ^d^	443.60 ± 10.25 ^e^

Data is represented by means ± standard deviations. SubCO_2_: Subcritical CO_2_ extraction; SFE: supercritical fluid extraction; Antioxidant: antioxidant activity using phosphomolybdenum complex; DPPH: DPPH free radical scavenging activity; FRAP: ferric reducing antioxidant power and ABTS: ABTS cation radical-scavenging. Means with small letter superscripts are significantly different (*p* < 0.05) within columns.

## Data Availability

The data presented in this study are available on request from the corresponding author.
